# Fungal Assemblages in Different Habitats in an Erman’s Birch Forest

**DOI:** 10.3389/fmicb.2016.01368

**Published:** 2016-08-30

**Authors:** Teng Yang, Huaibo Sun, Congcong Shen, Haiyan Chu

**Affiliations:** ^1^State Key Laboratory of Soil and Sustainable Agriculture, Institute of Soil Science, Chinese Academy of SciencesNanjing, China; ^2^University of Chinese Academy of SciencesBeijing, China; ^3^State Key Laboratory of Urban and Regional Ecology, Research Center for Eco-Environmental Sciences, Chinese Academy of SciencesBeijing, China

**Keywords:** fungal assemblages, habitat filtering, overlap and partition, phylogenetic relatedness, functional guild, Erman’s birch forest

## Abstract

Recent meta-analyses of fungal diversity using deeply sequenced marker genes suggest that most fungal taxa are locally distributed. However, little is known about the extent of overlap and niche partitions in total fungal communities or functional guilds within distinct habitats on a local forest scale. Here, we compared fungal communities in endosphere (leaf interior), phyllosphere (leaf interior and associated surface area) and soil samples from an Erman’s birch forest in Changbai Mountain, China. Community structures were significantly differentiated in terms of habitat, with soil having the highest fungal richness and phylogenetic diversity. Endophytic and phyllosphere fungi of *Betula ermanii* were more phylogenetically clustered compared with the corresponding soil fungi, indicating the ability of that host plants to filter and select their fungal partners. Furthermore, the majority of soil fungal taxa were soil specialists, while the dominant endosphere and phyllosphere taxa were aboveground generalists, with soil and plant foliage only sharing <8.2% fungal taxa. Most of the fungal taxa could be assigned to different functional guilds; however, the assigned guilds showed significant habitat specificity with variation in relative abundance. Collectively, the fungal assemblages in this Erman’s birch forest were strictly niche specialized and constrained by weak migration among habitats. The findings suggest that phylogenetic relatedness and functional guilds’ assignment can effectively interpret the certain ecological processes.

## Introduction

There are thought to be approximately 1.5 million fungal species on Earth, a conservative estimate based on the global-scale plant fungi ratio (1:6) (Hawksworth, 2001), yet much of this fungal diversity remains to be documented (Taylor et al., 2014). The recent rapid development of high-throughput sequencing tools has revolutionized the way fungal ecologists work (Peay, 2014), dramatically pushing the fungal species estimate to 5.1 million (Blackwell, 2011). For example, using 454 pyrosequencing, Zimmerman and Vitousek (2012) observed more than 4,200 fungal taxonomic units with 130 *Metrosideros polymorpha* samples across a Hawaiian landscape, while Cordier et al. (2012b) found a total of 3729 fungal operational taxonomic units (OTUs) in just 720 leaves of *Fagus sylvatica* along the French Pyrenees Mountains. Furthermore, Tedersoo et al. (2014) used pyrosequencing of 365 soil samples to perform a comprehensive global study of soil fungi, covering a total of 80,486 fungal OTUs.

Hyperdiverse fungal communities have also been discovered in various other habitats such as indoor human environments (Amend et al., 2010), wetlands and marine sediments (Wu et al., 2013; Redou et al., 2014), snail faces (O’Rorke et al., 2015), leaf litter (Voriskova and Baldrian, 2013), coral reefs (Amend et al., 2012), and even permafrost (Bellemain et al., 2013). However, although extremely high fungal diversity and ubiquity across various habitats is recognized, the variance in diversity and community structure among different habitats is not well characterized.

With their highly hierarchical structure, forests are typical representatives of a complex terrestrial ecosystem (Leiterer et al., 2015), providing independent partitions for both macroorganisms (Singleton, 2015) and microorganisms (Leff et al., 2015). Previous research suggests that fungal assemblages differ with depth in particular soil horizons, saprophytic fungi dominating the litter layer and mycorrhizal fungi the deeper soil profiles (Dickie et al., 2002; McGuire et al., 2013). In addition, fungal richness has also been shown to consistently decline with increasing soil depth (O’Brien et al., 2005). Studies on the spatial distribution of endophytic and phyllosphere fungi have further shown partitioned structures and spatial variability in aboveground parts of forest. For example, Scholtysik et al. (2013) found that infection density and fungal richness were higher in the understory than the crown, while sunexposed leaves in the top canopy exhibited the lowest infection rates. Moreover, Cordier et al. (2012a) observed dissimilarity between European beech phyllosphere fungal assemblages, which increased with distance between leaves within individual tree canopies, implying spatial autocorrelation of phyllosphere fungi on even a fine spatial scale. Nevertheless, direct comparisons between aboveground and belowground forest fungal assemblages using high-throughput amplicon sequencing remain limited (Coince et al., 2014; Meiser et al., 2014).

Aboveground and belowground fungal communities play vital roles in nutrient cycling (Osono, 2006; Averill et al., 2014) as well as contributing to forest health (Busby et al., 2016). Functional assignment of these fungal communities is therefore extremely important if we are to fully understand their ecology under different habitats (Nguyen et al., 2016). By analyzing fungal community datasets using functional guilds, soil fungal ecologists are able to effectively explain the biogeographic pattern and underlying process of specific guilds as well as the entire fungal community on a local (Bahram et al., 2016) and global scale (Tedersoo et al., 2014). Mycologists with interests in plant foliage may not be accustomed to analyzing fungal datasets by functional guilds, particularly when isolating fungi from surface-sterilized asymptomatic leaves (Arnold et al., 2007). Rather, they tend to use conventional or tacit assignment (fungal endophytes) (Wilson, 1995), even though fungal endophytes may have come from soil environments, switching among endophytic, pathogenic and saprotrophic lifestyles (Kuo et al., 2014). Here, we assigned all fungal taxa to five functional guilds: foliar endophytes, root endophytes, mycorrhiza, phytopathogens and saprophytes, to explore the characteristics of functional guilds in specific habitats.

Erman’s birch forest in Changbai Mountain, China, is a unique forest ecosystem located near the timberline and characterized by low temperatures, water stress and frost damage. It also harbors hyperdiverse fungal resources (Shen et al., 2014; Yang et al., 2016). Pure stands of *B. ermanii* grow at altitudes of 1820–2080 m (Bai et al., 2011), which is defined as well-protected subalpine forest in Northeast Asia (Bai et al., 2008). Our study area (∼1920 m a.s.l.) was located in the core zone of this forest stand. *B. ermanii* leaf and soil samples were collected simultaneously and fungal diversity, community structure, functional guilds, and phylogenetic relatedness in the endosphere, phyllosphere and corresponding soil analyzed. The following were examined: (1) whether or not distinct habitats have significantly different fungal diversity and community structure; (2) whether distribution of co-occurring species within each habitat (endosphere, phyllosphere and soil) in a community with respect to phylogeny is random or non-random (Webb et al., 2002); and (3) the degree of overlap in fungal communities in terms of taxonomic and functional groups among habitats. The findings will help advance our understanding of fungal ecology with respect to habitat on a neighborhood scale, and aid further analysis of the fungal impact on this unique temperate subalpine forest stand.

## Materials and Methods

### Study Area and Sampling

The study site was located in the core zone of Erman’s birch forest on the north slope of Changbai Mountain, Jilin province, Northeast China (42.058∼42.059°N, 128.065∼128.066°E; 1920 m a.s.l.). The mean annual temperature is -3.38°C and annual precipitation is 1053.2 mm/y. The soil is Permi-Gelic Cambosols, with a pH of 4.3–4.86 (Shen et al., 2013). The native forest consists of closed pure stands of *B. ermani* on an average slope of 13° (He et al., 2005). Although tourism flourished in the 1980s, the Changbai Mountain Nature Reserve, which was established in the early 1960s, now prohibits people from entering (Bai et al., 2008). The study area is therefore rarely affected by human disturbance and, in the absence of obvious environmental gradients and heterogeneous areas of vegetation, soil and topography; it is an exemplar natural subalpine deciduous forest ecosystem (Yu et al., 2014).

A sample transect (100 m × 100 m) was created on 30th August 2013 and 10 individual *B. ermanii* trees selected. Ten asymptomatic leaves were then collected from each tree to analyze foliar fungal endophyte communities. At the same time, six additional trees proximal to the above 10 were randomly selected for collection of a further 10 asymptomatic leaves to analyze phyllosphere fungal communities. Selected trees were located approximately 10 m apart. All leaves were sampled from the middle of different branches, from current-year branch shoots. The sample branches were oriented north, southeast and southwest. Leaves from each tree were collected in individual sterilized plastic bags and placed in coolers filled with ice packs.

Corresponding soil samples were collected from four independent replicate plots within the transect for analysis of soil fungal communities. Each plot (10 m × 10 m) was spaced 50 m apart. The specific procedure of soil sampling was as described previously (Shen et al., 2014); a schematic representation is shown in Supplementary Figure S1. All leaf and soil samples were brought back to the laboratory within 12 h and temporarily stored at 4°C until use.

### Sample Processing

Soil samples from each plot were homogenized together into a single sample and soil DNA extracted as described previously (Shen et al., 2013, 2014). Leaves were homogenized at the unit of individual trees. Leaf DNA was extracted using a Qiagen Plant DNeasy kit (Qiagen, Hilden, Germany) with minor modifications according to Zimmerman and Vitousek (2012). To distinguish between the two habitats, strict surface sterilization was carried out to target fungal endophytes (including the leaf surface and interior; Yang et al., 2016), while phyllosphere fungi were targeted without surface-sterilization (Cordier et al., 2012b).

Crude DNA of the soil and leaves were purified using a PowerClean Pro Clean-Up DNA kit (MO BIO Laboratories, Inc., Carlsbad, CA, USA) according to the manufacturer’s protocol. PCR systems and conditions were strictly consistent with the methods of Yang et al. (2016) for both soil and foliage. The internal transcribed spacer 1 (ITS1) region was selected for PCR amplification due to its applicability as a comparable fungal marker across forest soil (Buee et al., 2009), phyllosphere (Cordier et al., 2012b) and endosphere habitats (Yang et al., 2016). The forward primer (5′-CCATCTCATCCCTGCGTGTCTCCGAC TCAG NNNNNNN CTTGGTCATTTAGAGGAAGTAA-3′) contained the 454 Life Sciences primer A sequence, a four-base linker sequence (“TCAG”), a unique 7-bp barcode and fungal specific primer ITS1-F. The reverse primer (5′-CCTATCCCCTGTGTGCCTTGGCAGTC TCAG GCTGCGTTCTTCATCGATGC-3′) contained the 454 Life Sciences primer B sequence, a four-base linker sequence (“TCAG”) and fungal primer ITS2. Triplicate PCR products were pooled per sample to avoid PCR bias. After purification using an EasyPure Quick Gel Extraction Kit (TransGen Biotech, Beijing, China) and quantification with NanoDrop ND-1000 (Thermo Scientific, USA), the amplicons were sequenced on a Roche Genome Sequencer FLX System platform (454 Life Science, Branford, CT, USA). Standard Flowgram Format amplicon sequence data can be obtained from the sequence Read Archive (SRP048036) at the National Center for Biotechnology Information (NCBI).

### Bioinformatics and Statistics

Raw data from pyrosequencing were analyzed using the Quantitative Insights into Microbial Ecology (QIIME) pipeline 1.8.0 (Caporaso et al., 2010). The sequences were re-assigned to different samples using the *split_libraries.py* command with a minimum/maximum sequence length cutoff of 100/500 bp, and minimum average quality score allowed in the reads (>25). Sequences with homopolymers of >7 bp and any ambiguous base (N) or base pair mismatch in the primer regions were removed from the data set. Sequences were then denoised according to the method of Reeder and Knight (2010), resulting in 256,308 high quality sequences. ITSx 1.0.11^1^ was used to remove SSU, LSU, and 5.8S genes according to the Users’ guide (Bengtsson-Palme et al., 2013). This procedure deleted 3,471 sequences without any ITS1 regions detected.

The sequences were then clustered into OTUs at a 97% similarity threshold using the USEARCH algorithm (Edgar, 2010). Putative chimeric sequences were removed using a combination of *de novo* and reference-based Chimera checking with the flags *–non_chimeras_rentention = intersection* (Edgar et al., 2011). This procedure was carried out using the program UCHIME and implemented during the USEARCH clustering process. Taxonomy was assigned to fungal OTUs using the rdp option in the parallel_assign_taxonmy_rdp.py function with mini-confidence of 0.8 (Wang et al., 2007). The reference OTU database was obtained from the UNITE database (Version 7^2^). The “dynamic” representative/reference sequence file was used according to the recommendations of the manual (Koljalg et al., 2013).

Three representative sequences (putative OTUs) were not assigned to fungi, and were removed prior to subsequent analysis. Molecular singletons were removed from the downstream analysis to minimize the possibility of sequencing artifacts (Unterseher et al., 2011). Likewise, OTUs with less than 10 reads were removed from the analysis in line with recent research (Balint et al., 2015; Sapkota et al., 2015). By following this stringent quality filter and removing OTUs with low relative abundance resulted in more robust analysis. A total of 737 sequences were observed in one phyllosphere sample, and this minimum sequence sample was also removed. Finally, the total OTU matrix obtained was rarefied to 1733 sequences per sample (the minimum sequence among 19 samples) to compare the relative differences among samples.

A total of 479 OTUs existed in the un-subsampled OTU matrix, of which 64 were assigned to the fungal kingdom. We manually checked for a close match in the latest GenBank database with a basic local alignment tool (nblast, Altschul et al., 1990). According to the isolation sources, *e*-values, query coverage and identity of candidate sequences, we further specified 20 OTU assignments at least to the phyla level. Specific cutoff points and operations were determined according to the re-assignment of soil fungi by Tedersoo et al. (2014). They were then summarized with *summarize_taxa_through_plots.py script* in QIIME to determine the community structures in different habitats at the class level.

Richness, the chao1 index (Chao, 1984), good’s coverage (Good, 1953), evenness (Smith and Wilson, 1996) and Faith’s index of phylogenetic diversity (Faith’s PD; Faith, 1992) were used to compare fungal alpha diversities among different habitats. Faith’s PD contained the phylogenetic information, calculated using the picante package in R software (Kembel et al., 2010). Before calculating PD values, the representative sequences were aligned using the muscle algorithm (Edgar, 2004) and a Maximum-Likelihood Tree constructed using FastTree software (Price et al., 2010). Significant differences between diversity indices among the three habitats were tested with Duncan’s test at a confidence level of 95% using SPSS Statistics Version 20 (IBM SPSS, USA).

Distances between pairwise samples were calculated based on incidence, abundance and phylogenetic information, and betadiversity was shown with a cluster dendrogram using the function *hclust* in the R Stats Package^3^. For incidence information, we scaled the subsampled OTU table using a present/absent scale (0/1) using the function *decostand*, and calculated the Bray–Curtis distance with the vegan package (Oksanen et al., 2012). For abundance information, we directly calculated the Bray–Curtis distance (Bray and Curtis, 1957). For phylogenetic information, we used the mean nearest taxon distance (MNTD) separating OTUs into two communities to represent the phylo-betadiversity indices (Fine and Kembel, 2011; Stegen et al., 2012), which were calculated using Picante 1.6-2 (Kembel et al., 2010).

Significant differences in community assemblages among habitats were tested by permutational multivariate analysis of variance using distance matrices with the function *adonis* in the vegan package. In addition, OTUs and their relative abundances were randomized across the tips of phylogeny (null.model = ‘taxa.labels’, and abundance.weighted = True with the function *ses.mntd*) to evaluate the degree of non-random phylogenetic community structure. The standardized effect size measure (ses.MNTD) is used to quantify the number of standard deviations that the observed MNTD is from the mean of the null distribution (999 randomizations). The obtained ses.MNTD can be used to test for phylogenetic clustering or overdispersion (Webb et al., 2002). Negative *Z* values and low quantiles (*P* < 0.05) indicate that co-occurring species are more closely related than expected by chance (phylogenetic cluster). Conversely, positive values and high quantiles (*P* > 0.95) indicate that the co-occurring species are less closely related than expected by chance (overdispersion). Here, we also constructed phylogenetic trees of *Ascomycota* and *Basidiomycota*, respectively, in order to observe phylogenetic clusters or overdispersion within dominant phyla among habitats.

Finally, we merged all samples belonging to the same habitat, and rarefied the results to 35,328 sequences per habitat, which retained 479 OTUs (323 OTUs in the soil, 143 in the phyllosphere, and 148 in the endosphere). Based on the merged OTU Table, we created a network-like Venn diagram using Cytoscape 3.1.1 (Shannon et al., 2003). Core (abundant) and satellite (occasional or rare) OTUs with specific habitats were distinguished according to the method of Unterseher et al. (2011), and marked in the Venn diagram. Meanwhile, we summarized the relative abundances of the shared and exclusive fungal partitions among the three habitats, respectively, and assigned 479 OTUs to the functional guilds using a new open annotation tool (FUNGuild; Nguyen et al., 2016). Accordingly, a total of 190 OTUs (39.7%) were successfully assigned to functional guilds. The remaining OTUs were continually assigned to guilds referred to in the latest GenBank database with nblast, and the guilds’ information accepted if the identity and coverage was more than 97%. In addition, inference of ectomycorrhizal fungi was made with reference to a previous summary (Tedersoo et al., 2010; Branco et al., 2013). Finally, 307 OTUs were assigned to five functional guilds as follows: foliar endophytes, 68 OTUs; mycorrhiza, 103; pathogens, 45; root endophytes, 14; and saprophytes, 77. The sequence abundance of each partition in the venn diagram was subsequently summarized.

## Results

### General Characteristics of Fungal Communities in the Three Habitats

**FIGURE 1:**
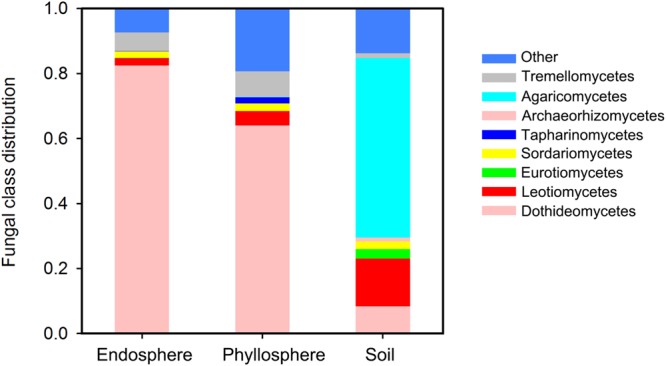
**Fungal class distribution among habitats.** Any classes accounting for less than 1% abundance are classified as “Others.”

The final data set comprised 243,359 high-quality reads covering 479 fungal OTUs. The mean number of reads per sample was 12,480 for the endosphere (range: 2,113–28,707), 7,065 for the phyllosphere (1,733–11,462), and 20,806 for the soil (9,732–30,071). At the phyla level, our dataset comprised 280 Ascomycota OTUs, 133 Basidiomycota, 1 Glomeromycota, 3 Rozellomycota, and 18 Zygomycota. Distinct community structures among habitats were observed at the fungal class level (**Figure 1**). Soil was dominated by Agaricomycetes (55.2% on average), Leotiomycetes (14.6%) and Dothideomycetes (8.4%). Phyllosphere and endosphere habitats showed a similarly high abundance of Dothideomycetes (82.4% in the endosphere and 64.1% in the phyllosphere), and Tremellomycetes (5.8 and 7.9%, respectively). Sordariomycetes accounted for ca. 2.2% in all three habitats, while Taphrinomycetes were phyllosphere specific (1.9%) and Eurotiomycetes soil specific (3.0%). At the phyla level, Glomeromycota, Rozellomycota and Zygomycota were found exclusively in the soil, albeit at low abundances.

The observed fungal OTU richness of the soil (*S*_obs_ = 129 ± 10) was significantly greater than that of the endosphere and phyllosphere (Duncan test, *P* < 0.05), but there was no significant difference in OTU richness between the endosphere and phyllosphere (**Table 1**; Supplementary Figure S2). The trend of Chao1, Good’s coverage and Faith’s PD values were all consistent with the results of OTU richness (**Table 1**).

**Table 1:** Comparison of alpha diversity indexes among habitats.

Habitat	Richness	Chao1	Good’s coverage	Evenness	PD
Endosphere	52 ± 12^b^	82 ± 16^b^	0.9873 ± 0.0024^a^	0.0597 ± 0.0217^a^	28 ± 6^b^
Phyllosphere	60 ± 13^b^	79 ± 14^b^	0.9893 ± 0.0028^a^	0.0789 ± 0.0824^a^	34 ± 7^b^
Soil	129 ± 10^a^	176 ± 14^a^	0.9750 ± 0.0009^b^	0.0767 ± 0.0348^a^	68 ± 8^a^

*Richness, observed number of OTUs; Chao1, Chao1 richness estimator; Good’s coverage, Good’s coverage estimator; Evenness, Simpson evenness; PD, Faith’s phylogenetic diversity index. Values represent means ± SD. Values in the same columns followed by different letters differ significantly at *P* ≤ 0.05 (Duncan’s test). The rarefaction of alpha diversity calculation was 1733 sequences (the minimum sequence among 19 samples).*

### Differentiation between Fungal Assemblages across Habitats

**FIGURE 2:**
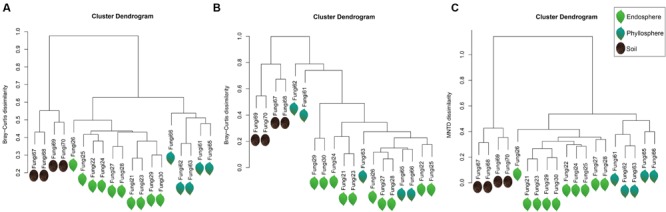
**Hierarchical clustering dendrogram of all samples. (A)** Bary–Curtis dissimilarity based on incidence data, **(B)** Bary–Curtis dissimilarity based on abundance data, and **(C)** MNTD dissimilarity based on phylogenetic data. The sequences were subsampled to 1733 sequences per sample. Fungi 21∼30 belong to the endosphere, and are represented by light green leaves; Fungi 61∼66 belong to the phyllosphere, and are represented by dark green leaves; and Fungi 67∼70 belong to the soil, and are represented by dark soil.

Fungal assemblages significantly differed among habitats (**Tables 2A,C**), except for weak differentiation between the endosphere and phyllosphere when using Bray–Curtis dissimilarity with abundance data (*P* = 0.086, **Table 2B**). Corresponding hierarchical clustering based on three betadiversity calculation types showed differentiation in terms of habitat (**Figure 2**). Based on phylogenetic MNTD matrices, the fungal phylo-assemblages were clearly divided into the three habitats with relatively high Pseudo F in ADONIS analysis (**Table 2C**). This suggests that differences in phylogenetic representation effectively drive the differentiation of fungal assemblages among habitats.

**Table 2:** Permutational multivariate analyses of variation of the compositional dissimilarity among habitats.

Multiple comparison	Pseudo *F*	*R*^2^	*P*
**(A)**
Endosphere vs. Phyllosphere vs. Soil	12.101	0.41582	0.001
Endosphere vs. Phyllosphere	6.547	0.33492	0.001
Endosphere vs. Soil	20.813	0.63429	0.001
Phyllosphere vs. Soil	12.408	0.63933	0.01
**(B)**
Endosphere vs. Phyllosphere vs. Soil	11.139	0.39587	0.002
Endosphere vs. Phyllosphere	2.1588	0.14241	0.086
Endosphere vs. Soil	18.798	0.61036	0.001
Phyllosphere vs. Soil	5.3786	0.43451	0.007
**(C)**
Endosphere vs. Phyllosphere vs. Soil	147.84	0.94563	0.001
Endosphere vs. Phyllosphere	7.4594	0.3646	0.001
Endosphere vs. Soil	54.826	0.82043	0.001
Phyllosphere vs. Soil	26.845	0.79317	0.001

***(A)** Bary–Curtis dissimilarity based on incidence data, **(B)** Bary–Curtis dissimilarity based on abundance data, and **(C)** MNTD dissimilarity based on phylogenetic data. Sequences were subsampled to 1733 sequences per sample.*

### Phylogenetic Relatedness within Habitats

**FIGURE 3:**
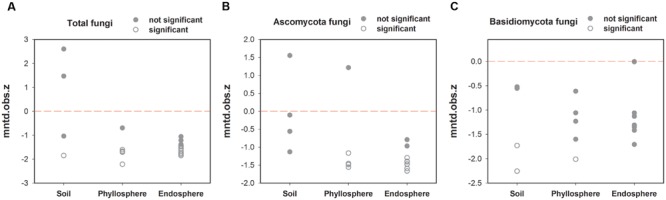
**Variation in standardized effect sizes of the mean nearest taxon distance (ses.MNTD) among habitats. (A)** Total fungi, **(B)** Ascomycota fungi, and **(C)** Basidiomycota fungi. “Not significant”: *P* > 0.05 and “significant”: *P* < 0.05.

The “standardized effect size” of MNTD can be used to observe differences between phylogenetic distances in observed local communities versus null communities generated with certain randomization methods. Here, with regards total fungal phylogenetic relatedness, the trend of most samples from the endosphere and phyllosphere was a phylogenetic cluster relative to that of the soil samples (**Figure 3A**). This trend was more obvious for Ascomycota fungi, which showed significantly lower *z* values in the majority of endosphere and phyllosphere samples (**Figure 3B**). This was interpreted to mean that co-occurring fungal taxa in the leaf interior and phyllosphere had smaller phylogenetic distances than expected. In contrast, no such phylogenetic trend was observed among habitats for the Basidiomycota fungi (**Figure 3C**).

### Habitat Overlap between Fungal Communities and Functional Guilds

**FIGURE 4:**
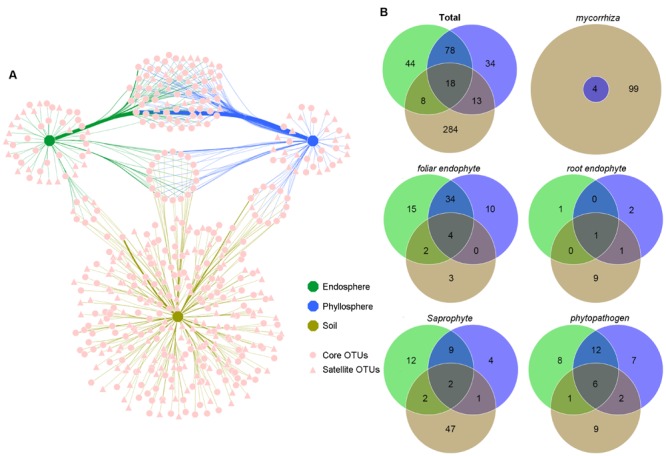
**Operational taxonomic unit (OTU) network map and venn diagrams of the different habitats (OTU numbers). (A)** OTU network map of total fungi communities across habitats. Each pink point represents an independent fungal OTU (479 OTUs; sequences were subsampled to 35,328 per habitat). Edges show which OTUs belonged to which Habitat. Edge thickness was weighted by the abundances from the OTU to the specific habitat. Different shaped points represent core or satellite OTUs in each habitat. **(B)** Venn diagrams of total fungal communities and five functional guilds among habitats. Green, blue and khaki represent samples belonging to the endosphere, phyllosphere and soil, respectively.

The network-like Venn diagram showed partitioning and overlap of the fungal OTUs among habitats. Accordingly, 280 core OTUs and 199 satellite OTUs were distinguished by their shape (**Figure 4A**). Unique OTUs were most abundant in the soil (284 OTUs, 80.94% of the total sequences), followed by 78 shared OTUs between the endosphere and phyllosphere (more than 80% of the total sequences in the endosphere and phyllosphere; **Figure 4B**; Supplementary Table S1). Pairwise OTUs coexisting in the endosphere and soil accounted for a tiny component of the sequences in both habitats, while pairwise OTUs coexisting in the phyllosphere and soil accounted for 18.26% of sequences in the soil samples, relative to 0.2% of sequences in the phyllosphere samples (Supplementary Table S1). Of note, 18 OTUs shared among the three habitats were all core OTUs, and showed particularly high abundance in the foliage samples (16% for the endosphere and 14.1% for the phyllosphere) relative to the soil samples (0.55% for the soil; Supplementary Table S1).

All five functional guilds were found in the soil environment; however, their distribution showed significant habitat specificity (**Figure 4B**; Supplementary Tables S2–S6). Mycorrhizal fungi existed exclusively in the soil. Although four OTUs were shared with the phyllosphere, their reads was only six in phyllosphere samples (Supplementary Table S4). Similarly, the majority of root endophytes existed in the soil (Supplementary Table S3). In contrast, most foliar endophytes and phytopathogens existed in the compartment shared between the endosphere and phyllosphere (Supplementary Tables S2 and S6), and most shared OTUs across habitats were phytopathogens and foliar endophytes, implying a complex life history and high dispersal capability. As expected, most saprophytes were found in the soil (Supplementary Table S5); however, 25 OTUs were found in the endosphere and phyllosphere, suggesting roles as pioneer decomposers of leaf litter of *B. ermanii*.

## Discussion

### High Diversity and Distinct Community Structures of Forest Fungi

At the regional scale, temperate forests in China harbor more diverse soil fungi than tropical and subtropical forests (Shi et al., 2014). Using 454 pyrosequencing, Buee et al. (2009) observed approximately 400 non-singleton fungal OTUs in six surface soil samples from a French temperate forest site, all of which had nearly identical levels of soil fungal diversity as the Erman’s birch forest. On the global scale, Tedersoo et al. (2014) found that the ratio of fungi-to-plant richness increased exponentially with increasing latitude, with high-latitude boreal forests supporting few tree and understory plant species but high soil fungal diversity (Wardle and Lindahl, 2014). In addition, Taylor et al. (2014) proposed that the fungus: plant ratio in *Picea mariana* forest soil from interior Alaska was at least 17:1. The Erman’s birch forest in our study belongs to an area of temperate subalpine forest, thus giving characteristics of temperate and boreal forest. Given that plant richness in such forests is approximately 20 species per plot on average (Shen et al., 2014), the fungus: plant ratio here is thought to be at least 16:1, corroborating the dominance of soil fungus at both high latitudes and in subalpine sites (Botnen et al., 2014; Timling et al., 2014).

Although soil had the highest fungal diversity, we found no significant differences in diversity between the endosphere and phyllosphere (**Table 1**, Supplementary Figure S2). This may be explained by the following: (i) high variance in fungal diversity among trees and among leaves, as previously reported for phyllosphere fungal assemblages of European beech (Cordier et al., 2012a); (ii) insufficient sampling and sequencing depth in the phyllosphere (Supplementary Figure S3); and/or (iii) deletion of OTUs with less than 10 reads prior to alpha diversity analysis. If singletons or low-abundance OTUs had been retained, significantly higher richness in the phyllosphere would have been observed relative to the endosphere (Supplementary Table S7).

In this study, the phyllosphere included the leaf interior and surface area; therefore, we don’t doubt that it harbors more fungal species than the endosphere. However, it is worth noting that the phyllosphere had greater variability than the endosphere with more singletons (Supplementary Table S7), perhaps representing incidental or rare phyllosphere species (Zimmerman and Vitousek, 2012). It should also be noted that even if some studies use the identical term “phyllosphere,” different preprocesses on leaves will recover different subsets of fungal communities associated with foliage (Jumpponen and Jones, 2009; Glenn et al., 2015; Sapkota et al., 2015).

Fungal community structures were also distinct among habitats (**Figure 1**). In soil, Ascomycota and Basidiomycota accounted for 91.3% of the total sequences, with 55.2% of sequences belonging to Agaricomycetes. This is consistent with the findings of Buee et al. (2009) who found Ascomycota and Basidiomycota to be the most dominant phyla, and Agaricomycetes the most dominant class in temperate forest soil. One reason for the similarity in soil fungal community structure between Buee et al. (2009) was that the same depth of sampled soil (0–5 cm) was used. However, in contrast, Dothideomycetes was the most dominant fungal class in the aboveground parts (82.4% in the endosphere and 64.1% in the phyllosphere). Previously, Kemler et al. (2013) found that foliar fungal endophytes of *Eucalyptus grandis* were dominated by Ascomycota, particularly by families in the Dothidiomycetes. This similar community structure was also reported by Zimmerman and Vitousek (2012) in a study of fungal communities in leaves of *Metrosideros ploymorpha*, and by Jumpponen and Jones (2009) in a study of fungal communities in temperate *Quercus macrocarpa* phyllosphere. However, the basic fungal community structure of tree leaves is quite different from that of bryophytes and lichens (Bates et al., 2011; Davey et al., 2013), indicating differences in host selection due to the remote phylogenetic distance and evolutionary history of plants (U’ren et al., 2010).

### Phylogenetic Relatedness Indicates Habitats

In this study, fungal phylogenetical assemblages were significantly segregated by the three habitat types based on MNTD distances (**Figure 2C, Table 2C**), although there was no significant difference between the endosphere and phyllosphere based on Bray–Curtis distance with abundance data (**Figure 2B, Table 2B**). Previously, Peay et al. (2010) proposed that phylogenetic betadiversity based on MNTD had its merits compared with traditional abundance-based betadiversity, especially when using multiple samples with a few shared OTUs. We created a phylogeny-based framework to examine whether the distribution of co-occurring species within habitats (or samples) in a community was non-random with respect to phylogeny. Using this framework with null models for community assembly can link ecological and evolutionary processes, allowing us to make inferences and test hypotheses on the dominant ecological process (Webb et al., 2002, 2006). Treseder et al. (2014) used 18S rRNA barcoded pyrosequencing to determine large-scale soil fungal distribution with evolutionary history, and found support for the tropical conservatism hypothesis; that is, older fungal phyla preferring significantly lower latitudes and warmer, wetter conditions than younger phyla. Furthermore, Liu et al. (2015) found that AM fungal communities in an alpine meadow were phylogenetically clustered in unfertilized soil, random under low-fertilizer treatment and overdispersed under high-fertilizer treatment, implying that the dominant ecological process shifted from habitat filter to a stochastic process and finally to competitive exclusion along the fertilizer gradient. Here, we found a positive “standardized effect size” of MNTD (*z* values) in the majority of soil samples (0.95 > *P* > 0.05), indicating the dominant role of a stochastic process in this local forest soil. Similar results were previously reported for a tropical ectomycorrhizal fungal community, which was assembled randomly with respect to phylogeny in sand soil types (Peay et al., 2010). Nevertheless, this result is in contrast to most previous studies on soil bacteria, all of which show phylogenetic clustering in soil bacterial communities (Stegen et al., 2012; Wang et al., 2013; Shen et al., 2015). Differences in dispersal capability, adaption to environmental stresses, rates of horizontal gene transfer, and phylogenetic supertree construction between bacteria and fungi may lead to this distinction in terms of phylogeny (Peay et al., 2016).

It is also possible that interspecific competition between ectomycorrhizal, ericoid mycorrhizal fungi, root endophytes and saprophytic fungi from the litter layer might break the local environmental filter in soil fungal communities (McGuire et al., 2013). In contrast, however, the majority of endosphere and phyllosphere samples exhibited significantly negative *z* values (*P* < 0.05), suggesting that co-occurring fungal taxa in these two habitats had smaller phylogenetic distances than expected (phylogenetic cluster). Assuming that the symbiotic trait with the host plant is phylogenetically conserved, the observed phylogenetic cluster will show the increased importance of plant filtering during the long-term co-evolution of plants and fungi (Saunders and Kohn, 2009).

### High Habitat Specificity and Low Overlap of Total Fungi and Functional Guilds

Erman’s birch forest is a simple subalpine forest ecosystem, which presents plants with extreme cold and water stress (Yu et al., 2014). Annually, the deciduous habit of *B. ermanii* not only creates periodical nutrient dynamics, but also continuous input of leaf-associated fungi to the soil fungal pool, usually enhancing niche overlap. However, the species co-existence theory lends more support to niche partition (specificity), with co-existing species occupying different niches, thus minimizing competition for limited resources and strengthening the division of labor, which facilitates habitat partitioning (Webb et al., 2002). Talbot et al. (2014) found that most soil fungi were endemic to particular bioregions across North American, while Meiser et al. (2014) proposed limited shared fungal OTUs among different environments through meta-analysis of deep-sequenced fungal studies. Here, the majority of soil fungal taxa were soil specialists (284 OTUs accounting for 80.94% of soil sequences), while dominant fungal taxa of the endosphere and phyllosphere were generalists, coexisting on aboveground parts (78 OTUs accounting for more than 80% of sequences in the endosphere and phyllosphere, respectively). Soil and aboveground parts of *B. ermanii* shared <8.2% fungal taxa. The most dominant soil fungus was assigned to *Amanita* sp. C42 EC252 (a common ectomycorrhizal fungi in forest soil), which accounted for 23.48% of the total sequences in soil.

Previously, *Sebacina* and *Russula* were found to be dominant ECMF of *B. ermanii* in a volcanic desert on Mount Fuji, Japan (Nara, 2006), while an investigation in Northeast China showed that *Amanita* was also a dominant ECMF associated with *B. ermanii* (Meng and Shao, 2001). In the phyllosphere and endosphere, the most dominant fungus was assigned to *Sphaerulina* sp. CBS 128758 with high coverage and identity (100%). This species was also previously isolated from *Lysimachia clethroides* as a foliar endophyte in South Korea. Its relative abundance accounted for more than half of the total fungal sequences aboveground, indicating a strong association with *B. ermanii* foliage in this location. A recent systematic mycological study showed that *Sphaerulina* can cause leaf spots on many important trees and herbs such as *Rubus fruticosus, Zelkova serrata*, poplar and *Acer* (Verkley et al., 2013). Potential host jumping between *B. ermanii* and other species and conversion of this genus between endophytism and pathogenicity should therefore be considered in future studies (Chaverri and Samuels, 2013; Xu et al., 2014).

Notably, although eight OTUs were shared between the soil and endosphere, and 13 between the soil and phyllosphere, the distribution of abundance per habitat was distinct (Supplementary Table S1). Those shared between the soil and endosphere were of low abundances, while those shared between the soil and phyllosphere were more abundant in the soil (18.26% vs. 0.2%, respectively). This is thought to be the result of random spread from the soil to the leaf surface; that is, fungi in this compartment assigned more to mycorrhizal fungi and root endophytes (Supplementary Tables S3 and S4). A total of 18 fungal OTUs coexisted in all habitats; however, they were more abundant in the endosphere and phyllosphere than the soil (16, 14.1 and 0.55%, respectively). Moreover, based on functional guild information (Supplementary Tables S2 and S6), we inferred that most were phytopathogens and foliar endophytes, which may end up in the soil via fallen leaves.

In addition, a considerable proportion of soil fungi were designated ECMF (Supplementary Table S4), corroborating their dominance in *Betula* monodominant stands (Nara and Hogetsu, 2004; Nara, 2006; Deslippe and Simard, 2011). We also found some root endophytes in the soil (Supplementary Table S3), which are frequently reported to coexist with ECM plants in cold environments (Bjorbaekmo et al., 2010; Blaalid et al., 2014; Botnen et al., 2014). The reads distribution of the particular functional guilds suggested obvious high habitat specificity. High relative abundances in aboveground parts were observed for foliar endophytes and phytopathogens, while high reads in the soil belonged to mycorrhizal fungi, root endophytes and saprophytes. Collectively, habitat specificity dominated fungal assemblages in this local forest stand, especially considering the assignment of specific functional guilds. Meanwhile, there was weak habitat overlap among environmental fungal communities due to stochastic dispersal and fallen leaves.

## Conclusion

In summary, we reported, for the first time, a significant difference in fungal communities in three different habitats (leaf endosphere, phyllosphere and soil) in an Erman’s birch forest. Soil was found to contain high fungal diversity despite low plant diversity; however, fungal communities associated with the foliage were found to be more phylogenetically clustered than those in the soil habitats. This implies the strong niche filter effects of host plants. Consistent with prominent fungal endemism on a large scale, limited fungal taxa were shared among different habitats at the local scale. Particularly in terms of specific functional guilds, habitat specificity rather than habitat overlap dominated their assemblages, implying that despite convenient dispersal at the local scale and fungal species input with annual falling leaves, an environmental filter based on different habitat characteristics is the dominant force behind local fungal assemblages in this location.

## Author Contributions

TY did the sampling work, performed the experiments, analyzed the data and drafted the manuscript. HS helped in the related sampling, experiments and data analysis. CS helped in the related sampling and participated in the revision of the manuscript. HC conceived the study, supervised the research and revised the manuscript.

## Conflict of Interest Statement

The authors declare that the research was conducted in the absence of any commercial or financial relationships that could be construed as a potential conflict of interest.

## Footnotes

1 http://microbiology.se/software/itsx/

2 http://unite.ut.ee

3 http://cran.stat.sfu.ca/
