# Supplementary material for: Fungal Assemblages in Different Habitats in an Erman’s Birch Forest
Source: Front Microbiol. 2016 Aug 30;7:1368. doi: 10.3389/fmicb.2016.01368 (PMC5003828; doi:10.3389/fmicb.2016.01368)
Supplement: Supplementary file 1 [file Data_Sheet_1.DOC]

**Supplementary information**

Table S1 Fungal abundance distribution of overlap among habitats

| Percentage of abundance | | Habitat | | |
| --- | --- | --- | --- | --- |
|  |  | Endosphere | Phyllosphere | Soil |
| Unique | | 3.21% | 4.33% | **80.94%** |
| Pairwise | Endosphere ~ Phyllosphere | **80.64%** | **81.37%** | - |
|  | Endosphere ~ Soil | 0.14% | - | 0.25% |
|  | Phyllosphere ~ Soil | - | 0.20% | 18.26% |
| All | | 16.00% | 14.10% | 0.55% |

*Numbers in bold indicate the dominant fungal partitions in each habitat.

Table S2 Reads distribution of overlap among habitats for foliar endophytes

| Reads number | | Habitat | | |
| --- | --- | --- | --- | --- |
|  |  | Endosphere | Phyllosphere | Soil |
| Unique | | 763 | 755 | 22 |
| Pairwise | Endosphere ~ Phyllosphere | **23,172** | **26,503** | - |
|  | Endosphere ~ Soil | 8 | - | 13 |
|  | Phyllosphere ~ Soil | - | 0 | 0 |
| All | | 916 | 3057 | 143 |

*Numbers in bold indicate the preferred habitat where a large number of foliar endophytes were found. Sequences were subsampled to 35,328 per habitat.

Table S3 Reads distribution of overlap among habitats for root endophytes

| Reads number | | Habitat | | |
| --- | --- | --- | --- | --- |
|  |  | Endosphere | Phyllosphere | Soil |
| Unique | | 4 | 26 | **343** |
| Pairwise | Endosphere ~ Phyllosphere | **0** | **0** | - |
|  | Endosphere ~ Soil | 0 | - | 0 |
|  | Phyllosphere ~ Soil | - | 1 | **492** |
| All | | 1 | 1 | 143 |

*Numbers in bold indicate the preferred habitat where a large number of root endophytes were found. Sequences were subsampled to 35,328 per habitat.

Table S4 Reads distribution of overlap among habitats for mycorrhizal fungi

| Reads number | | Habitat | | |
| --- | --- | --- | --- | --- |
|  |  | Endosphere | Phyllosphere | Soil |
| Unique | | 0 | 0 | **19,999** |
| Pairwise | Endosphere ~ Phyllosphere | 0 | 0 | - |
|  | Endosphere ~ Soil | 0 | - | 0 |
|  | Phyllosphere ~ Soil | - | 6 | **5235** |
| All | | 0 | 0 | 0 |

*Numbers in bold indicate the preferred habitat where a large number of mycorrhizal fungi were found. Sequences were subsampled to 35,328 per habitat.

Table S5 Reads distribution of overlap among habitats for saprophytes

| Reads number | | Habitat | | |
| --- | --- | --- | --- | --- |
|  |  | Endosphere | Phyllosphere | Soil |
| Unique | | 211 | 64 | **3725** |
| Pairwise | Endosphere ~ Phyllosphere | 62 | 160 | - |
|  | Endosphere ~ Soil | 31 | - | 52 |
|  | Phyllosphere ~ Soil | - | 4 | 12 |
| All | | 7 | 138 | 12 |

*Numbers in bold indicate the preferred habitat where a large number of saprophytes were found. Sequences were subsampled to 35,328 per habitat.

Table S6 Reads distribution of overlap among habitats for phytopathogens

| Reads number | | Habitat | | |
| --- | --- | --- | --- | --- |
|  |  | Endosphere | Phyllosphere | Soil |
| Unique | | 93 | 466 | 439 |
| Pairwise | Endosphere ~ Phyllosphere | **4734** | **1625** | - |
|  | Endosphere ~ Soil | 31 | - | 18 |
|  | Phyllosphere ~ Soil | - | 16 | 6 |
| All | | **2430** | **1315** | 25 |

*Numbers in bold indicate the preferred habitat where a large number of phytopathogens were found. Sequences were subsampled to 35,328 per habitat.

Table S7 Comparison of richness and singletons between the endosphere and phyllosphere

(a)

| Habitat | Richness | Singletons |
| --- | --- | --- |
| Endosphere | 40±11 | 18±4 |
| Phyllosphere | 58±16 | 30±9 |
| Independent t-test *P* value | 0.012 | 0.004 |

(b)

| Habitat | Richness | Singletons |
| --- | --- | --- |
| Endosphere | 39±11 | 18±4 |
| Phyllosphere | 55±14 | 26±7 |
| Independent t-test *P* value | 0.02 | 0.009 |

Richness: the observed number of OTUs. Singletons: the number of OTUs accounting for only one read in the sample. Values represent means ± SD. (a) Singletons included in the total OTUs. The rarefaction of alpha diversity calculation was 752 sequences (n_endosphere_=10, n_phyllosphere_=6). (b) Singletons excluded from the total OTUs. The rarefaction of alpha diversity calculation was 748 sequences (n_endosphere_=10, n_phyllosphere_=6).


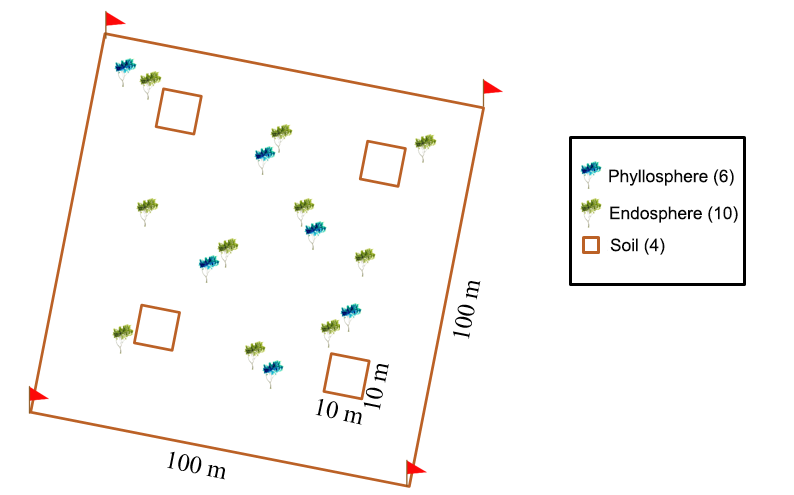


Fig. S1 A schematic chart showing the sampling framework in Erman’s birch forest.


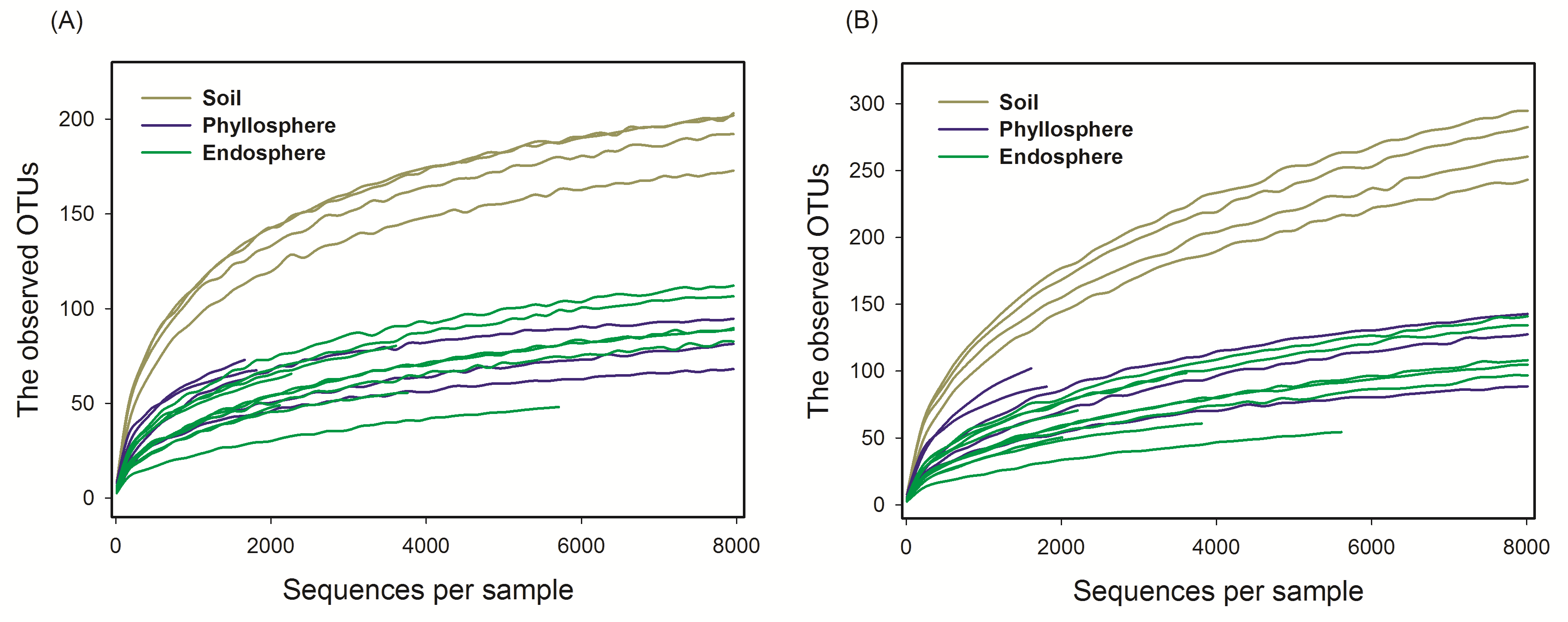


Fig. S2 Rarefaction curves of the observed OTUs with sequence depth based on OTU clustering at 97% sequence identity for the ITS1 region. (A) OTUs with less than 10 reads removed; (B) only singletons removed.


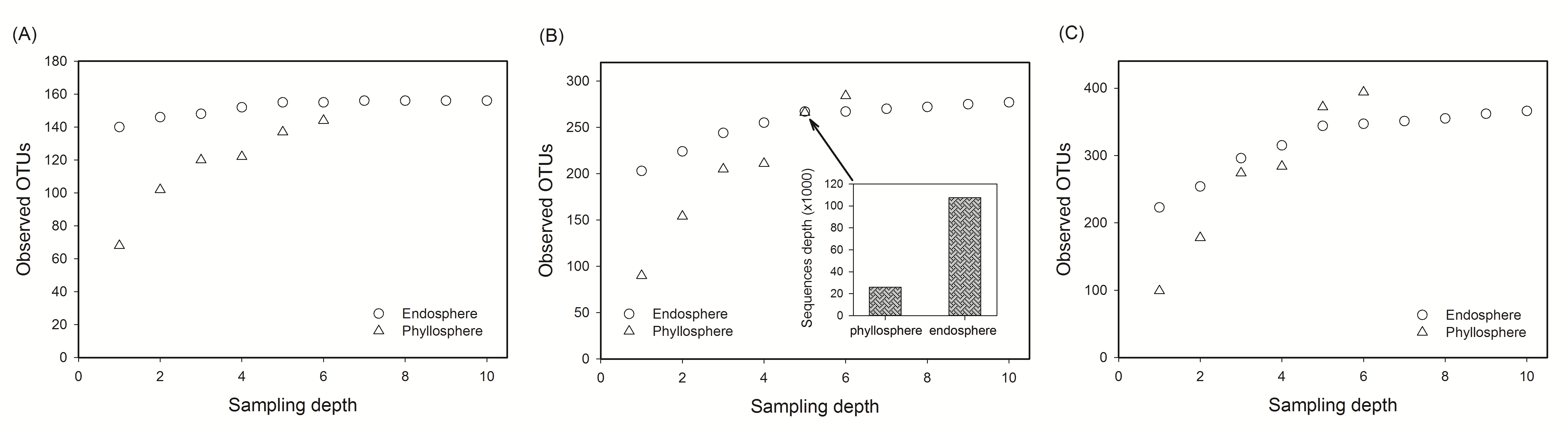


Fig. S3 Rarefaction curves of the observed OTUs with sampling depth, based on OTU clustering at 97% sequence identity for the ITS1 region in endosphere and phyllosphere samples. (A) OTUs with less than 10 reads removed from the total OTUs; (B) singletons removed from the total OTUs; (C) Singletons retained in the total OTUs.
